# Supplementary material for: Organisation and delivery of liaison psychiatry services in general hospitals in England: results of a national survey
Source: BMJ Open. 2018 Sep 1;8(8):e023091. doi: 10.1136/bmjopen-2018-023091 (PMC6120655; doi:10.1136/bmjopen-2018-023091)
Supplement: Supplementary file 1 [file bmjopen-2018-023091supp001.pdf]

On-line appendices for paper entitled "The Organisation and Delivery of Liaison Psychiatry Services in General Hospitals in England: results of a National Survey".

Appendix 1: Questions used in the Second National Liaison Psychiatry Survey (LPSE-2) in 2015.

<u>Questions 1-10: Location</u>

1. What is the name of your Liaison Psychiatry service (if it has one)?
2. What is the name of the Acute Hospital(s) you are based in?
3. What is the name of the Acute Trust(s) you are based in?
4. Does the Acute Trust(s) have more than one site with inpatient beds? If so, please name them.
5. Does the Acute Trust(s) have more than one A&E? If so, please name them.
6. Does your Liaison Psych service provide services to all the sites?
7. If not, can you give us a contact details of the other liaison psychiatry service(s) please?
8. What is the provider of your service? (Usually this is the mental health trust)
9. Is psych liaison in your Acute Trust provided by one or many providers? If many, which?

If the above questions do not capture details of your service, please explain here:

<u>Questions 11-12: Target population</u>

10. What services do you provide, and to whom? (Some only see self-harms, some see anyone in the whole hospital, others are in-between. Some look after alcohol problems, some not, some do LD, some not, etc.) What are the age-criteria for your service(s)?
11. Do you support anything other than the acute care pathway? Are there any clinics, etc. If so, can you outline the nature of the work?

<u>Questions 13-18: Staffing</u>

12. Number of FTE nurses and their bands (if working age adults and older adults are separate services, please collate these separately)
13. Number of FTE doctors and their grades (if working age adults and older adults are separate services, please collate these separately).
14. Number of FTE admins and their grades (if working age adults and older adults are separate services, please collate these separately).
15. Number of other clinicians and their grades if known (if working age adults and older adults are separate services, please collate these separately).
16. Number of other non-clinicians and their grades if known (if working age adults and older adults are separate services, please collate these separately).
17. Of the above, who is substantive and who is a locum, part of winter pressures. fixed term appointments, etc?

<u>Questions 19-20: funding</u>

18. What is your service's budget, if known? (Leave out the medics (or just junior medics) if necessary).
19. How much of that that budget is permanent and how much is temporary (if known)?

<u>Questions 21-23: Mental health service context</u>

20. What are your service's hours of operation? (Out Of Hours SHO cover does not mean your service is 24/7).
21. Does your service do all the work contained in all the referrals? (eg is some passed on to other services? Please explain)

    (This question is about things like requests for psych opinions from wards, which are sometimes passed straight on to the duty SHO)
22. Are there other mental health workers in your acute trust who are not part of your service? (eg counsellors, psychologists)

<u>Questions 24-28: Commissioning context</u>

23. Have you undertaken any research (published or not) to support the development of your service? If so, can you describe it please?
24. Is your service better resourced than it was a year ago? If so, how? If worse, please also explain.

25. If the services are separate, how do people transfer from CAMHS to Working Age Adults and from Working Age Adults to Older Persons?
(This is usually age cut-offs plus exceptions and complications. There seems to be huge variety in this and we would like to catalogue it.)

26. Does your service have a response time standard and is that time agreed with referrers and/or commissioners?

Appendix 2: Original and Modified criteria for describing Rapid Access Intervention and Discharge (RAID) services

Original RAID definition	• 24 hours, 7 days a week<br>• Age inclusive; no separate Older Age Adult or Working Age Adult teams<br>• Response targets of 1 hour to Emergency Department, 24 hours to wards<br>• Multidisciplinary team<br>• Comprehensive; see referrals for all clinical problems<br>• Brief follow-up clinics
Modified RAID definition	• 24 hours, 7 days a week<br>• Age inclusive; no dedicated Older Age Adult or Working Age Adult service<br>• Multidisciplinary team<br>• Response targets of 1 hour to Emergency Department , 24 hours to wards<br>• Either, not comprehensive (e.g. do not see substance misuse or self-harm referrals) or no follow-up clinics

Appendix 3: Core classifications according to Aitken et al, 2014. (13)

SubCore	Less Than Core
Core	2 consultants, 0.6 other medical,<br>2 band 7 nurses,<br>6 band 6 nurses,<br>0 other therapists<br>1 band 7 team manager,<br>0.2 band 8 clinical services manager<br>2.6 admins<br>9-5 hours<br>Sees everyone aged 16+
Core24	2 consultants, 2 other medical<br>6 band 7 nurses<br>7 band 6 nurses<br>4 other therapists<br>1 band 7 team manager<br>0.2-0.4 band 8 clinical services manager<br>2 admins<br>1 business support<br>24/7<br>Special older adults<br>Special Drugs and alcohol
Enhanced	4 consultants, 2 other medical<br>3 band 7 nurses<br>7 band 6 nurses<br>2 other therapists<br>1 band 7 team manager<br>0.2-0.4 band 8 clinical services manager<br>2 admins<br>1 business support<br>24/7<br>Special older adults<br>Special Drugs and alcohol<br>Outpatient services.
Comprehensive	5 consultants, 2 other medical<br>2 band 8b nurses<br>17 band 6 nurses<br>10 band 5 nurses<br>16 other therapists<br>3 band 7 team manager

	1 band 8 clinical services manager<br>12 admins<br>1 business support<br>24/7<br>Special older adults<br>Special Drugs and alcohol<br>Outpatient services<br>Specialties
