# Supplementary material for: Organisation and delivery of liaison psychiatry services in general hospitals in England: results of a national survey
Source: BMJ Open. 2018 Sep 1;8(8):e023091. doi: 10.1136/bmjopen-2018-023091 (PMC6120655; doi:10.1136/bmjopen-2018-023091)
Supplement: Author's manuscript [file bmjopen-2018-023091.draft_revisions.pdf]

BMJ Open is committed to open peer review. As part of this commitment we make the peer review history of every article we publish publicly available.

When an article is published we post the peer reviewers' comments and the authors' responses online. We also post the versions of the paper that were used during peer review. These are the versions that the peer review comments apply to.

The versions of the paper that follow are the versions that were submitted during the peer review process. They are not the versions of record or the final published versions. They should not be cited or distributed as the published version of this manuscript.

BMJ Open is an open access journal and the full, final, typeset and author-corrected version of record of the manuscript is available on our site with no access controls, subscription charges or pay-per-view fees (http://bmjopen.bmj.com).

If you have any questions on BMJ Open's open peer review process please email info.bmjopen@bmj.com

BMJ Open

The Organisation and Delivery of Liaison Psychiatry Services in General Hospitals in England: results of a National Survey

Journal:	*BMJ Open*
Manuscript ID	bmjopen-2018-023091
Article Type:	Research
Date Submitted by the Author:	20-Mar-2018
Complete List of Authors:	Walker, Andrew; National Institute of Health Research Clinical Research Network<br>Barrett, Jessica; Royal College of Psychiatrists, National Collaborating Centre for Mental Health<br>Lee, William; University of Plymouth, Peninsula Schools of Medicine & Dentistry<br>West, Robert; University of Leeds, Leeds Institute of Health Sciences<br>Guthrie, Elspeth; University of Leeds Leeds Institute of Health Sciences, Psychological and Social Medicine<br>Trigwell, Peter; Leeds and York Partnership NHS Foundation Trust, National Inpatient Centre for Psychological Medicine<br>Quirk, Alan; Royal College of Psychiatrists, CCQI<br>Crawford, Mike; Imperial College London, Psychological Medicine<br>House, Allan; Academic Unit of Psychiatry,
Keywords:	consultation-liaison, liaison psychiatry, hospital psychiatry, development

SCHOLARONE™
Manuscripts

For peer review only

The Organisation and Delivery of Liaison Psychiatry Services in General Hospitals in England: results of a National Survey

Walker A[1]. A, Barrett J.R[2], Lee W[3], West R[4], Guthrie E[4], Trigwell P[5], Quirk A[6], Crawford M.J [6,7], House A[4]

1. Clinical Research Network National Coordinating Centre, National Institute of Health Research Clinical Research Network, Leeds
2. National Collaborating Centre for Mental Health, The Royal College of Psychiatrists
3. Institute of translational and Stratified Medicine, Plymouth University Peninsula Schools of Medicine and Dentistry. Plymouth. UK.
4. Leeds Institute of Health Sciences, University of Leeds. Leeds. UK.
5. National Inpatient Centre for Psychological Medicine, Leeds and York Partnership NHS Foundation Trust. Leeds. UK.
6. College Centre for Quality Improvement. Royal College of Psychiatrists, London. UK.
7. Centre for Psychiatry. Department of Medicine. Imperial College London. London. UK.

Corresponding author: Professor Else Guthrie: e.a.guthrie@leeds.ac.uk

Key words: Consultation-liaison, development, hospital psychiatry, liaison psychiatry

Word Count :3943

Abstract: 298

For peer review only - http://bmjopen.bmj.com/site/about/guidelines.xhtml

ABSTRACT

Objectives: To describe current provision of hospital-based liaison psychiatry services in England, and to determine different models of liaison service that are currently operating in England.

Design: Cross-sectional observational study comprising an electronic survey followed by targeted telephone interviews.

Setting: All 179 acute hospitals with an Emergency Department in England.

Participants: 168 hospitals that had a liaison psychiatry service completed an electronic survey. Telephone interviews were conducted for 57 hospitals that reported specialist liaison services additional to provision for acute care.

Measures: Data included the location, service structures and staffing, working practices, relations with other mental health service providers, policies such as response times and funding. Model-2based clustering was used to characterise the services. Telephone interviews identified the range of additional liaison psychiatry services provided.

Results: Most hospitals (141, 79%) reported a 7-day service responding to acute referrals from the Emergency Department and wards. However, under half of hospitals had 24-hour access to the service (78, 44%). A third of hospitals (57, 32%) provided non-acute liaison work including outpatient clinics and links to specialist hospital services. 156 hospitals (87%) had a multidisciplinary service including a psychiatrist and mental health nurses. We derived a four-cluster model of liaison psychiatry using variables resulting from the electronic survey; the salient features of clusters were staffing numbers, especially nursing; provision of rapid response 24-hour 7-day acute services; offering outpatient and other non-acute work, and containing age-specific teams for older adults.

Conclusions: This is the most comprehensive study to date of liaison psychiatry in England, and demonstrates the wide availability of such services nationally. Although all services provide an acute assessment function, there is no uniformity about hours of coverage or expectation of response times. Most services were better characterised by the model we developed than by current classification systems for liaison psychiatry.

For peer review only - http://bmjopen.bmj.com/site/about/guidelines.xhtml

For peer review only

Article Summary

Strengths and limitations of this study

- A comprehensive national survey of liaison psychiatry services in acute hospitals, at a time of increased government investment, and debate about equity of access to mental and physical healthcare.
- The survey obtained 100% response rate for all hospitals in England with an Emergency Department.
- Classification of services was carried out using model-based clustering.
- A limitation was that service provision was reported by the services themselves rather than based on independent observation.
- Mental health services provided by clinicians outside of the liaison psychiatry service were not comprehensively reviewed.

For peer review only

INTRODUCTION

Liaison psychiatry is the sub-specialty of psychiatry concerned with clinical practice, teaching and research in non-psychiatric clinical settings: the ‘liaison’ referred to is therefore between psychiatry and other clinical disciplines. In the UK it has been largely based in acute (“general”) hospitals. The origins of liaison psychiatry can be traced to the 1930s but substantial growth only occurred in the post-war decades (1-4): The UK’s Royal College of Psychiatrists established its Faculty of Liaison Psychiatry in 1997 and first published a competency-based curriculum for postgraduate training in Liaison Psychiatry in 2009 (5).

The case for liaison psychiatry services rested initially upon observations that the prevalence of many psychiatric problems in acute hospitals is well above general population levels and that such co-morbidities can pose particular management challenges (6). People with problems such as psychosis, panic, delirium or self-harm may present to the Emergency Department, or their difficulties may become apparent on inpatient wards - perhaps requiring rapid assessment and intervention (7). Liaison psychiatry services also see people with more longstanding problems such as difficulty adjusting to severe physical illness, or complex physical health and mental health conditions – such work mostly being undertaken in outpatient clinics.

Recent interest in liaison psychiatry in the UK (8) has been focussed on two issues – cost savings that might result from the service, and the need to provide equitable access to emergency care for all patients regardless of whether their problems are primarily physical, psychiatric, or a combination (9).

The suggestion that financial savings from timely psychiatric intervention are sufficient to pay for the liaison psychiatry service undertaking that intervention, the so-called “cost-offset” effect, is not new (10). Most recently it attracted interest in the UK following the publication of a report from one English hospital which reported that their “Rapid Assessment Intervention and Discharge” (RAID) service achieved reductions in average inpatient lengths of stay in the target population of up to four days, even for patients not directly seen by the service (11) (12).

An important influence in current debate in the UK has been the classification of hospitals in terms of four service grades proposed by Aitken et al 2014 (13) (See Appendix for details). This classification was based on services already in existence that were capable of delivering certain levels of coverage in the hospital. It has been used to inform commissioning of services, with the aim

For peer review only - http://bmjopen.bmj.com/site/about/guidelines.xhtml

that all hospitals with Emergency Departments should have a liaison service meeting such standards by 2020 (14).

Here we present findings from the most detailed survey of liaison psychiatry services yet undertaken in England, describing their structures and staffing levels and their relation to other mental health services associated with the acute hospitals in which they are located. The aim was to describe current provision of hospital-based liaison psychiatry services in England, and to determine different models of liaison service that are currently operating.

This work arises from the first phase of a programme of research funded by the National Institute of Health Services (NIHR), to evaluate the cost-effectiveness and efficiency of different configurations of liaison psychiatry services in England (LP-MAESTRO) (http://www.nets.nihr.ac.uk/projects/hsdr/135808), and an annual mapping survey of liaison services funded by Health Education England, NHS England and the Royal College of Psychiatrists. A prior survey of liaison psychiatry services was carried out in 2013 (unpublished), and this paper describes the second study (15), which was carried out in conjunction with the LP-MAESTRO programme.

METHOD

Setting and sample

The sample consisted of all acute hospitals in England that had an Emergency Department at the time. Acute Trusts were identified from the NHS website (www.nhs.uk/servicedirectories/pages/nhstrustlisting.aspx) and individual hospitals were then identified from Trust websites.

Within each hospital liaison psychiatry service we identified *components* of service - typically defined by the part of the hospital covered by that component - for example: emergency department, ward referrals, links to specialist services, liaison psychiatry outpatient clinics.

Each component of the service might then have different *characteristics* such as staff mix, working hours, performance targets, patient-groups seen etc.

Design

Cross-sectional two-stage survey conducted by email and telephone interview.

Measures

The email survey ran between 14 May 2015 and 30 April 2015. The survey was brief and allowed flexible (free text) responses. Response was by email or telephone. Non-responding hospitals and missing response items were followed up by email and telephone. The questions asked in the email are given in Appendix 1.

We derived two variables describing RAID services. The first, “original RAID”, is based upon the description provided in Tadros et al (12) of the service evaluated at Birmingham City Hospital; the second, “modified RAID”, is based upon the profile of current services in Birmingham still known as RAID. We characterised each service according to whether they met the criteria for either of these

For peer review only - http://bmjopen.bmj.com/site/about/guidelines.xhtml

service types. We also used responses on staffing level or working practice to classify each service according to recent guidance from NHS England that was created to help commissioners in planning service delivery (13). The grades used in the guidance are as follows: Comprehensive (full liaison provision), Enhanced 24 (staffed according to the original RAID model), Core 24 (provides acute provision for a hospital with an Emergency Department, but no out-patient work) and Core (intended for less busy hospitals); services not meeting Core criteria were classified as Sub-Core (see Appendices 2 and 3 for details).

A telephone interview survey ran between the 16.7.15 and 30.9.15. It was undertaken to obtain further details about services that reported that they provided liaison services in addition to provision for acute care of patients in the ED or on the acute hospital wards (e.g. out-patient services or specialist renal input).

Data from the survey have been published by the Royal College of Psychiatrist (15). What we present in this paper is a re-classification of these data (carried out by the LP-MAESTRO team), a statistical analysis of the data using cluster analysis, and results of the telephone survey, none of which has been previously published.

Patient involvement

Further work in the LP-MAESTRO programme will focus on patient experience.

ANALYSIS

The main analyses were undertaken with R statistical software version 3.2.2 (R core team 2016).

A latent class model (16) was fitted to perform clustering of responding hospitals. The number of clusters to be used was determined by minimising the Bayesian Information Criterion (BIC) because the BIC tends to favour less complexity. Models were fitted only if the number of observations (168) exceeded the number of parameters used in the model, thus ensuring a positive number of degrees of freedom. Other hospital properties were extracted from the survey and used as covariates in this model-based clustering approach.

Many of the variables used in clustering were categorical. Variables which might have been regarded as continuous were categorised so that all were handled in a similar way. For example, the number of hours of operation of the service was defined as three categories: 40–80 hours per week, 81–167 hours per week, and 168 (=7x24) hours per week. Since all variables to be clustered were categorical, the polytomous latent class analysis package poLCA version 1.4.1 (16) with R statistical software version 3.2.0 (R core team 2015) was used for all analyses. The latent class function made use of the Expectation–Maximisation algorithm and there was the possibility of convergence to a local maximum rather than a global maximum. To overcome this, multiple starts were used (17).

RESULTS

Staffing and working practices

For peer review only - http://bmjopen.bmj.com/site/about/guidelines.xhtml

Data were obtained on all 179 acute hospitals identified in England: 168 (94%) reported that they had a Liaison Psychiatry service; eleven had no service. All 168 hospitals with a liaison service completed the electronic survey and answered questions in follow up emails and telephone calls, ensuring that there were no missing data.

Twelve services were nurse-only services. All other services were multidisciplinary and all included at least a psychiatrist of some grade and a mental health nurse. One hundred and forty one hospitals (79%) reported at least one consultant psychiatrist as part of the team (total number =195), 95 hospitals (53%) reported other psychiatrists (non-consultant grade), 42 hospitals (23%) reported a psychologist or psychological therapist as part of the team, 26 hospitals (15%) reported allied health professionals and 52 hospitals (29%) reported other mental health staff. All 168 hospitals with a liaison service had nursing staff as part of the team and there were 1,384 whole time equivalents working in liaison services at the time of the survey.

141 hospitals (79%) provided a 7-day service and 15 hospitals (8%) provided a service Monday to Friday. Of the 141 hospitals providing 7-day services, 78 (55%) reported a 24-hour 7-day service.

Out of the 168 hospitals that had a liaison service, 75 hospitals (45%) had target response times of one hour or less for referrals from the Emergency Department and 73 hospitals (43%) reported target response times to referrals from the wards as less than one day. Sixty four hospitals (38%) had no target response time.

Nearly all of the liaison services (99%) saw patients who were referred following self-harm (167 hospitals) and many saw patients for assessment of alcohol and substance misuse (106 hospitals 63%). Only 37 services (22%) saw patients with learning disabilities. 44 services (26%) had separate older adult and working age adult teams.

57 hospitals (34%) reported a service or component of service that did more than serve the acute care pathway, and 4 of these hospitals operated virtually separate liaison services for acute and non-acute referrals.

Classification according to RAID and Core.

Of the 168 hospitals, only 8 met the original RAID criteria and 35 met criteria for modified RAID. Ten liaison services had the term RAID in their title, without meeting either of the RAID criteria.

Of the 168 hospitals, one was rated as Comprehensive, three were Enhanced24 (2%), 13 were Core24 (8%), 18 were Core (11%) and 133 were Sub-Core (79%). The Comprehensive rated service met modified RAID criteria, two of the Enhanced24 services met Original RAID criteria and the final Enhanced24 service did not meet either RAID criterion. Of those services that met either RAID criterion, 28/41 (68%) were rated as Core or Sub-Core.

Types of liaison psychiatry service: results from cluster analysis

We used data from the email survey to cluster the services using characteristics listed in Table 1.

The minimum value of Bayesian Information Criterion was with four clusters, but the value for three clusters was very near the minimum also. Hence a decision was required between three and four clusters and we decided the model with four clusters was more interpretable and useful. Hospitals

For peer review only - http://bmjopen.bmj.com/site/about/guidelines.xhtml

were assigned to a cluster according to their modal probability: that is hospitals were labelled as a certain cluster when the model gave a probability of membership of that cluster to be larger than that of any other. Table 2 shows the modal cluster membership tabulated against hospital characteristics.

Model-based clustering identified four classes. These do not represent discrete categories but rather services that are relatively similar to each other in a diverse landscape.

- Cluster 1: Services tended to be based in smaller hospitals, had the smallest numbers of consultant staff and nurses. Only a minority offered 24-hour 7-day cover, few had predefined response times and none met either of the RAID criteria. Few offered outpatient clinics and none offered care outside the acute pathway.
- Cluster 2: Services were most likely to meet one of the RAID criteria, providing 24-hour 7-day cover, working to response-time targets for Emergency Department and ward referrals and concentrating exclusively on the acute care pathway with no follow-up outpatient clinics.
- Cluster 3: Services were more diverse with some offering 24-hour 7-day services, but the defining feature, was that they also offered outpatient clinics and covered care outside the acute care pathway; they had the highest number of consultants and nurses – number of nurses being an important determinant of the probability of membership in this cluster.
- Cluster 4: These were also diverse services, a third offering outpatient clinics and work outside the acute pathway; only a minority provided 24-hour 7-day cover or worked to response time targets and none met either of the RAID criteria. All these hospitals had separate teams for working-age adults and for older persons.

The nature of clinical services; telephone interviews

We undertook telephone interviews covering 57 hospitals and 61 separate liaison services; four hospitals had two distinctly different liaison teams. The telephone interviews reflected the clustering with most of those interviewed being in clusters 3 and 4: cluster 1: n=8, cluster 2: n=4, cluster 3: n=30 and cluster 4: n=19.

Emergency Department referrals

57 out of the 61 services (93%) saw acute referrals from the Emergency Department. Most (53, 87%) were available Monday to Sunday, and n=32 (52%) were available 24 hours a day. 49 (80%) of the services responded to referrals of adult patients of any age, but entry criteria could be quite specific – for example, one service saw all working age adults throughout the day and older age adults only for the first half of the night. Most Emergency Department liaison psychiatry teams (51, 84%) were multidisciplinary although five consisted of nursing staff only.

Referrals from the Emergency Department varied considerably in scope and numbers; out of the 46 reported referral rates the mean number of weekly referrals was 36 (min=1, max=100). In addition to assessment, most services that were interviewed (36, 59%) offered Emergency Department patients out-patient follow-up.

All of the 57 services which served the Emergency Department had key performance indicators, and almost three in four (n=43, 70%) measured patient outcome in some way.

For peer review only - http://bmjopen.bmj.com/site/about/guidelines.xhtml

Ward referrals

Fifty seven services accepted ward referrals. Most (n=46, 75%) were available to wards 7 days, and nearly one third (n=20, 33%) were available to wards 24 hours a day. Almost three out of four (n=44, 72%) responded to referrals from wards for adult patients of any age, five services responded to referrals from wards only for older adults, and nine responded to referrals from wards only for working age adults.

Again most ward teams (53, 87%) were multidisciplinary teams although four consisted of medical staff only, three consisted of nursing staff only, and one staffed the ward team with psychiatrists and psychologists. Based upon 47 reported referral rates the mean number of weekly referrals was 25. All responding services assessed patients and offered short term follow-up on medical wards, and over half (n=34, 56%) offered out-patient follow-up.

50 of those we interviewed had key performance indicators for wards, and 42 (69%) measured patient outcome in some way for ward referrals.

Self-harm referrals

All but two services accepted referrals for self-harm. 51 (84%) offered a 7 day service, 29 (48%) provided a service 24 hours a day, and most (n=46, 75%) offered a service to adults of all ages.

Most services we interviewed (54, 89%) said their self-harm teams were multidisciplinary. All services assessed patients on wards and approximately half (n=31, 51%) offered short-term follow-up on medical wards. Only eight services described a separate self-harm out-patient clinic, although several services described seeing small numbers of selected patients.

Liaison psychiatry with named specialist services

Twenty services (33%) provided specialist liaison services to at least one named specialist service or department in the hospital. A total of 31 different specialist services were reported; the most frequently reported were gastroenterology (n=5), hepatology (n=4) palliative care (n=4), maternity, neurology, trauma and transplant (n=3 each).

Outpatient clinics

33 services (54%) provided a general liaison psychiatry outpatient clinic and 28 services (46%) reported running an outpatient clinic for particular specialist groups; 20 services had both types of clinic. Twenty two of the general liaison psychiatry clinics saw patients of any adult age and 11 clinics saw working age adults only. Thirteen clinics were staffed with a multidisciplinary team, 15 were solely medical, two had nursing staff only and three clinics included a psychologist. Referrals to the general liaison psychiatry clinic came predominantly from the acute hospital in which they were based (n=26). Thirty one clinics offered short-term treatment and follow-up and 18 offered longer-term treatment and follow-up.

The most commonly reported specialist clinics were for medically unexplained symptoms (n=7), diabetes (n=4), bariatric surgery patients (n=4), respiratory disease (n=3) and perinatal psychiatry (n=3). Most clinics offered some form of psychological therapy – Problem Solving Therapy, (n=46,

For peer review only - http://bmjopen.bmj.com/site/about/guidelines.xhtml

75%) Motivational Interviewing, (n=38, 62%) Cognitive Behavioural Therapy (n=35, 57%) Behavioural Activation (n=31, 51%) or Interpersonal Therapy (n=26, 43%).

Other acute hospital mental health providers

In order of frequency, 47 liaison services (77%) co-existed in the acute hospital with separate drug or alcohol services, 44 (72%) with clinical psychology, and 22 (36%) co-existed with health psychology. We identified a wide range of other services – for particular patient groups or for overlapping patient groups by other agencies.

Referral to local service providers

The ease of referral to other mental health services for patients requiring follow up was also investigated. All services said they could routinely refer to a local community mental health team, 52 (85%) could refer to a crisis team routinely, 54 (89%) could refer routinely to drug and or alcohol and the same number to older adult psychiatry. Over half (n=36, 59%) of services could routinely refer to clinical psychology and 19 (31%) to health psychology.

Non-clinical activity

All services we interviewed provided some form of non-clinical work in the form of staff training or educational sessions, medico-legal assessments, advice to managers and others. The most common non-clinical services were: dementia training (n=17); research and service evaluation (n=16); organizational support and advice to acute hospital staff (n=15); delirium training (n=11); Mental Capacity Act training (n=10).

Discussion

We identified widespread availability of liaison psychiatry services in acute hospitals in England. Liaison psychiatry teams were customarily multidisciplinary and most services saw all acute mental health problems in the hospital and adults of all ages. Our findings suggest that there has been a gradual but continued expansion in liaison psychiatry services over the last 20 years, as evidenced by several previous surveys including those focusing on consultant posts in the British Isles (18,19), services in a particular area of England (20) and the one previous unpublished national survey undertaken at the request of NHS-England (LPSE-1). As an example of the expansion, the number of consultant posts in liaison psychiatry in the British Isles more than doubled from 43 in 1998 (18) to 93 in 2003 (19). The findings of the current survey suggest a further increase with 195 consultant posts in liaison psychiatry in England alone.

We found 11 hospitals that reported having no liaison service at all, which is concerning given that one of the targets set by NHS England in the Five Year Forward View for Mental Health is that by 2020 no acute hospitals should be without all-age mental health liaison services in emergency departments and inpatient wards (21).

For peer review only - http://bmjopen.bmj.com/site/about/guidelines.xhtml

For peer review only

Only a third of services offered outpatient clinics and non-acute care. The range of such activities was wide, with more than 30 different specialist services. There was however very little in common between services about which specialist activities were supported. Surprisingly few services (just over 10% of our total sample) reported running clinics that supported longer-term follow up and treatment opportunities, a *sine qua non* for the management of problems with living with long-term illness or of severe and chronic medically unexplained symptoms (22). This gap in service provision was most striking in self-harm services; we found that the majority of services offered acute assessment but no service offered routine therapeutic treatment for service users.

Very few of the services we surveyed readily fitted into the current commissioning framework (13), or the RAID framework, so the classification into Core, Core24, Enhanced24, or Comprehensive had limited value in discriminating between hospitals, and neither descriptive framework proved useful in identifying those services that reported 24-hour 7-day acute services.

For these reasons we sought a more practical, data-driven approach to describing service types. We chose model-based clustering to do so. Alternative approaches would have been to use one of many heuristic algorithms such as hierarchical clustering, k-means, self-organising maps, graph-theoretic approaches, or support vector machines. A generative mixture model has the advantage that it can be more general and provides a statistical framework within which to decide upon the number of clusters present. Model-based clustering has also been found to perform better than other approaches in identifying clusters (16). The models were simply parameterised since there were only 168 observations. Within a diverse picture of provision, our cluster analysis did reveal some patterns of service – the three most obvious features that distinguished between services were the hours of cover and response time standards, the likelihood of providing non-acute care in outpatients, and the decision to have separate teams for older and working age adults. Size of hospital and staffing levels (especially nursing) were important associations with the type of service offered. This suggests that when services scale up from the basic provision represented by Cluster 1 (and found in smaller hospitals) they do so in one of these three directions – increasing intensity of acute work, developing outpatient and non-acute work, or developing specialist old age teams.

Our findings have implications for those commissioning and those providing services.

First, we found widespread availability of liaison psychiatry services in English acute hospitals, but most teams were poorly resourced compared to published recommendations. Second, whatever local decisions are made about liaison psychiatry, our survey suggests national co-ordination of services is lacking. Third, we were struck by the unexpectedly low levels of longer-term outpatient treatment provision. Problems of adjustment to long-term illness, persistently poor adherence to challenging treatment regimes, medically unexplained symptoms and severe somatoform disorders all form part of the *raison d'etre* for liaison psychiatry and their management requires sustained professional input, in the hospital as well as in community settings. Our results confirm previous findings about the low national level of provision for people who harm themselves.

There are several limitations to our study. Our approach to surveying provided a rather general high-level account of services that doesn't do full justice to the richness and diversity of provision in multi-component services. Reliance on a single (or occasionally a second) informant at each stage

For peer review only - http://bmjopen.bmj.com/site/about/guidelines.xhtml

may have led to missing or inaccurate information. The service descriptors we used were based on self-report, and we have not verified them with direct independent observation. Our sampling strategy meant we did not collect information on specialist hospitals without Emergency Departments, so we did not collect data on rare but important facilities in specialist hospitals. Our survey was entirely hospital focused and while we are aware of (and involved in) initiatives to develop and evaluate primary care-based liaison psychiatry services, they were not studied here.

There is increasing interest in the idea that well-run liaison psychiatry services can be both important in improving quality of care in acute hospitals and cost-effective (23-25). UK liaison services are changing rapidly, with a round of investment especially in provision for emergency assessment and response (26). A further national survey of all English acute hospitals has been completed and the results will be published in the near future. It is hoped this latest survey will provide further coverage of a rapidly changing landscape.

Liaison psychiatry services in the UK are being encouraged by their specialty representative group in the Royal College of Psychiatrists to use a standardised package of outcome measures, the Framework for Routine Outcome Measurement in Liaison Psychiatry (FROM-LP) (27), to enable benchmarking against national norms. On the basis of these and other evaluation exercises we expect to achieve an increasingly detailed and nuanced account of the nature and impact of liaison psychiatry – a subspecialty that has a valuable role in providing genuinely co-ordinated and inclusive healthcare.

For peer review only

For peer review only - http://bmjopen.bmj.com/site/about/guidelines.xhtml

Acknowledgments: This work was supported by the efforts of S Keane, M Heneghan, S Chee, J Lewis, S Jayakumar at the Royal College of Psychiatrists and the NIHR Clinical Research Network (CRN) Yorkshire and Humber.

Funding: The present study arises from two funding initiatives: A national survey of staffing and structure in liaison psychiatry services in acute hospitals completed on behalf of the Royal College of Psychiatrists and the National Collaborating Centre for Mental Health commissioned by NHS England (Liaison Psychiatry Survey of England 2015, LPSE 2015) and a research study funded by the UK's National Institute for Health Research HS&DR programme to evaluate the effectiveness and cost-effectiveness of liaison psychiatry services (LP-MAESTRO project number 13/58/08).

Department of Health Disclaimer: The views and opinions expressed therein are those of the authors and do not necessarily reflect those of the HS&DR, NIHR, NHS or the Department of Health.

Author Contributions: AH, MJC, EG, and PT conceived of the research. JRB and WL carried out the survey. AW co-ordinated the telephone interviews. RW carried out the statistical analysis. All authors contributed to the manuscript and approved the final version.

Provenance and peer review: Not commissioned; externally peer reviewed.

Competing Interests: All authors have completed the ICMJE uniform disclosure form at http://www.icmje.org/coi_disclosure.pdf and declare: no support from any organization for the submitted work; no financial relationships with any organizations that might have an interest in the submitted work in the previous three years, no other relationships or activities that could appear to have influenced the submitted work.

Ethical approval and consent

Return of the email survey was taken as implicit consent. NHS Ethical permission (REC reference: 15/NS/0025) and Trust level approval was obtained for the telephone interviews. Telephone survey interviewees provided verbal consent at the start of the interview.

Data Sharing

Survey data are available from the author WL.

For peer review only

References

1. Lipowski Z. Consultation-liaison psychiatry: the first half century. General hospital psychiatry. 1986;8(5):305-15.
2. Mayou R. The history of general hospital psychiatry. The British Journal of Psychiatry. 1989;155(6):764-76.
3. Schwab JJ. Consultation-liaison psychiatry: a historical overview. Psychosomatics. 1989;30(3):245-54.
4. Aitken P, Lloyd G, Mayou R, Bass C, Sharpe M. A history of liaison psychiatry in the UK. BJPsych Bull 2016; 40: 199-203.
5. The Royal College of Psychiatrists. A competency-based curriculum for specialist training in psychiatry: sub-specialist module in liaison psychiatry. RCPsych London. 2006.
6. Bourgeois J HD, Servis M, Hales R. Consultation-liaison psychiatry: advantages for healthcare systems. Disease Management and Health Outcomes. 2005;13(2):93-106.
7. Academy of Medical Royal Colleges. Managing Urgent Mental Health needs in the Acute Hospital: a guide for practitioners, managers and commissioners. London 2009.
8. Joint Commissioning Panel for Mental Health.. Guidance for commissioners of liaison mental health services to acute hospitals. London. 2012.
9. Parsonage M, Fossey M, Tutty C. Liaison psychiatry in the modern NHS. London: Centre for Mental Health. 2012.
10. Strain JJ, Hammer JS, Fulop G. APM task force on psychosocial interventions in the general hospital inpatient setting: a review of cost-offset studies. Psychosomatics. 1994;35(3):253-62.
11. Parsonage M, Fossey M. Economic evaluation of a liaison psychiatry service: Centre for Mental Health London; 2011.
12. Tadros G, Salama RA, Kingston P, Mustafa N, Johnson E, Pannell R, et al. Impact of an integrated rapid response psychiatric liaison team on quality improvement and cost savings: the Birmingham RAID model. The Psychiatrist Online. 2013;37(1):4-10.
13. Aitken P, Robens S, Emmens T. Developing Models for Liaison Psychiatry Services-Guidance. Strategic Clinical Network for Mental Health, Dementia and Neurological Conditions South West. England 2014.
14. Department of Health, England. Achieving Better Access to Mental Health Services by 2020. London. 2014.
15. Barrett J, Aitken P, Lee W. Report of the 2nd Annual Survey of Liaison Psychiatry in England. Royal College of Psychiatrists https://www.rcpsych.ac.uk/pdf/CALC_LIA2015WilliamLee1.pdf Retrieved Dec 12 2017

16. Linzer DA, Lewis JB. poLCA: An R package for polytomous variable latent class analysis. Journal of Statistical Software. 2011;42(10):1-29.

17. Yeung KY, Fraley C, Murua A, Raftery AE, Ruzzo WL. Model-based clustering and data transformations for gene expression data. Bioinformatics. 2001;17(10):977-87.

18. Guthrie E Development of liaison psychiatry: Real expansion or a bubble that is about to burst. BJPsych Bulletin 1998; 22:291-293.

19. Swift G, Guthrie E. Liaison psychiatry continues to expand: developing services in the British Isles. BJPsych Bulletin 2003; 27: 339-341.

20. Ruddy R, House A. A standard liaison psychiatry service structure? The Psychiatrist. 2003;27(12):457-60.

For peer review only - http://bmjopen.bmj.com/site/about/guidelines.xhtml

21.Mental Health Taskforce Strategy. The Five Year Forward View for Mental Health. A report from the independent Mental Health Taskforce to the NHS in England. Feb 2016 www. nhs.uk/mentalhealth/taskforce

22. Creed FH, van der Feltz-Cornelius C, Guthrie E, Henningsen P, Rief W, Schroder A, White P. Identification, assessment and treatment of individual patients. Chapter in Medically Unexplained Symptoms, Somatisation and Bodily Distress: Developing better clinical services. Eds F Cree, P Henningsen & p Fink. Cambridge University Press. 2011 p175-216.

23. Wood R, Wand AP. The effectiveness of consultation-liaison psychiatry in the general hospital setting: a systematic review. Journal of psychosomatic research. 2014;76(3):175-92.

24. de Araujo Andreoli PB, de Albuquerque Citero V, de Jesus Mari J. A systematic review of studies of the cost-effectiveness of mental health consultation-liaison interventions in general hospitals. Psychosomatics. 2003;44(6):499-507.

25. Hosaka T, Aoki T, Watanabe T, Okuyama T, Kurosawa H. General hospital psychiatry from the perspective of medical economics. Psychiatry and clinical neurosciences. 1999;53(4):449-53.

26. NHS England . Guidance to support the introduction of access and waiting time standards for mental health services in 2015/16. https://www.england.nhs.uk/wp-content/uploads/2015/02/mh-access-wait-time-guid.pdf Retreived 10 Dec 2017.

27. Trigwell P, Kustow J, Santhouse A, et al. *Framework for Routine Outcome Measurement in Liaison Psychiatry (FROM-LP).* Faculty Report FR/LP/02. Royal College of Psychiatrists, 2015.

For peer review only - http://bmjopen.bmj.com/site/about/guidelines.xhtml

Table 1: The characteristics derived from survey responses used to distinguish Liaison Psychiatry services in the model based clustering

**Labelling**	1. Does the name of the service include 'RAID'?<br>2. Is the service classified as Sub-core, Core, or does it meet one of the definitions Core 24, Enhanced, or Comprehensive?<br>3. Does the service operate 7 days per week, or for less than 7 days?<br>4. How many hours per week is the service provided?
**Coverage**	5. Does the service claim to cover all mental health?<br>6. Is there a dedicated working-age adults (18–65) team?<br>7. Is there a dedicated older adults (65+) team?
**Work done**	8. Does the service undertake work from the Emergency Department?<br>9. Does the service undertake in-reach work?<br>10. Does the service operate an out-patient clinic?<br>11. Does the service have pathways other than acute pathways?<br>12. What is the response time for the Emergency Department?<br>13. What is the response time for the wards?
Other aspects of hospitals (Note that for some variables, the value assigned may have been inferred from other survey responses rather than taken from the direct response given.)	14. Number of services within a hospital (1,2, or 3)<br>15. Number of providers of services (1 or 2)<br>16. Number of hospital beds<br>17. Number of nurses employed by the liaison psychiatry service<br>18. Number of consultants<br>19. Number of services<br>20. Number of service providers
Additional variables	21. Does the service meet the original RAID criteria?<br>22. Does the service meet the modified RAID criteria?

RAID = Rapid Assessment Intervention and Discharge

For peer review only - http://bmjopen.bmj.com/site/about/guidelines.xhtml

For peer review only

Table 2: Hospital characteristics according to cluster membership

Hospital characteristic	Cluster 1 N=46	Cluster 2 N=35	Cluster 3 N=43	Cluster 4 N=44
RAID				
Name has RAID in title	1 (2%)	7 (20%)	10 (23%)	1 (2%)
Not codable for RAID	3 (6%)	1 (3%)	5 (12%)	1 (2%)
Not RAID	43 (94%)	11 (31%)	20 (46%)	43 (98%)
Original RAID	0 -	0 -	6 (14%)	0 -
Modified RAID	0 -	23 (66%)	12 (28%)	0 -
Core classification				
Sub-Core	45 (98%)	23 (66%)	29 (67%)	36 (82%)
Core	1 (2%)	5 (14%)	7 (16%	5 (11%)
Core 24	0 -	7 (20%)	3 (7%)	3 (7%)
Enhanced24	0 -	0 -	3 (7%)	0 -
Comprehensive	0 -	0 -	1 (2%)	0 -
Service operates 7 days	33 (72%)	34 (97%)	34 (79%)	40 (91%)
Hours of Operation				
40–80 hours	15 (33%)	1 (3%)	8 (19%)	15 (34%)
81–167 hours	16 (34%)	7 (20%)	13 (30%)	15 (34%)
7x24 = 168 hours	15 (33%)	27 (77%)	22 (51%)	14 (31%)
Serves all MH	32 (70%)	21 (60%)	26 (60%)	19 (43%)
Dedicated WAA	0 -	0 -	0 -	44 (100%)
Dedicated OAA	0 -	0 -	0 -	44 (100%)
OP clinic	2 -	0 -	43 (100%)	14 (32%)
Non-acute pathway	0 -	0 -	43 (100%)	14 (32%)
Response time to the ED				
<1h	3 (6%)	35 (100%)	25 (58%)	12 (27%)
1.5–4h	17 (37%)	0 -	4 (9%)	11 (25%)
Not stated or >4h	26 (57%)	0 -	14 (33%)	21 (48%)
Response time to wards				
<24h	8 (17%)	29 (83%)	23 (53%)	13 (30%)
36h–5d	8 (17%)	6 (17%)	0 -	8 (18%)
Not stated	30 (65%)	0 -	20 (46%)	23 (52%)
Hospital beds				
50–447	19 (41%)	14 (40%)	12 (28%)	8 (18%)
447–621	20 (43%)	8 (24%)	16 (37%)	16 (36%)
622–1943	7 (15%)	13 (54%)	17 (40%)	20 (45%)
Number of FTE nurses				
0.5–6.0	25 (54%)	8 (23%)	11 (26%)	16 (36%)
6.1–9.4	15 (33%)	11 (31%)	11 (26%)	15 (34%)
9.5–24.0	6 (13%)	16	21 (49%)	13 (30%)
Number of FTE consultants				
0.0–0.5	27 (57%)	7 (20%)	9 (21%)	15 (34%)
0.6–1.4	16 (35%)	13 (37%)	12 (28%)	13 (30%)
1.5–9.2	3 (6%)	15 (43%)	22 (51%)	16 (36%)
Single service provider	45 (98%)	33 (94%)	41 (95%)	40 (91%)
Two service providers	1 (2%)	2 (6%)	2 (5%)	4 (9%)
Number of services within same hospital				
One	44 (96%)	35 (100%)	39 (91%)	27 (61%)
Two	1 (2%)	0 -	4 (9%)	16 (36%)
Three	1 (2%)	0 -	0 -	1 (2%)

MH Mental Health WAA= Working Age Adults OAA =Older Adults Service FTE= Full time equivalent

For peer review only - http://bmjopen.bmj.com/site/about/guidelines.xhtml

Appendix 1: Questions used in the Second National Liaison Psychiatry Survey (LPSE-2) in 2015.

Questions 1-10: Location

1. What is the name of your Liaison Psychiatry service (if it has one)?
2. What is the name of the Acute Hospital(s) you are based in?
3. What is the name of the Acute Trust(s) you are based in?
4. Does the Acute Trust(s) have more than one site with inpatient beds? If so, please name them.
5. Does the Acute Trust(s) have more than one A&E? If so, please name them.
6. Does your Liaison Psych service provide services to all the sites?
7. If not, can you give us a contact details of the other liaison psychiatry service(s) please?
8. What is the provider of your service? (Usually this is the mental health trust)
9. Is psych liaison in your Acute Trust provided by one or many providers? If many, which?

If the above questions do not capture details of your service, please explain here:

Questions 11-12: Target population

10. What services do you provide, and to whom? (Some only see self-harms, some see anyone in the whole hospital, others are in-between. Some look after alcohol problems, some not, some do LD, some not, etc.) What are the age-criteria for your service(s)?
11. Do you support anything other than the acute care pathway? Are there any clinics, etc. If so, can you outline the nature of the work?

Questions 13-18: Staffing

12. Number of FTE nurses and their bands (if working age adults and older adults are separate services, please collate these separately)
13. Number of FTE doctors and their grades (if working age adults and older adults are separate services, please collate these separately).
14. Number of FTE admins and their grades (if working age adults and older adults are separate services, please collate these separately).
15. Number of other clinicians and their grades if known (if working age adults and older adults are separate services, please collate these separately).
16. Number of other non-clinicians and their grades if known (if working age adults and older adults are separate services, please collate these separately).
17. Of the above, who is substantive and who is a locum, part of winter pressures. fixed term appointments, etc?

Questions 19-20: funding

18. What is your service's budget, if known? (Leave out the medics (or just junior medics) if necessary).
19. How much of that that budget is permanent and how much is temporary (if known)?

Questions 21-23: Mental health service context

20. What are your service's hours of operation? (Out Of Hours SHO cover does not mean your service is 24/7).
21. Does your service do all the work contained in all the referrals? (eg is some passed on to other services? Please explain)

    (This question is about things like requests for psych opinions from wards, which are sometimes passed straight on to the duty SHO)
22. Are there other mental health workers in your acute trust who are not part of your service? (eg counsellors, psychologists)

Questions 24-28: Commissioning context

23. Have you undertaken any research (published or not) to support the development of your service? If so, can you describe it please?
24. Is your service better resourced than it was a year ago? If so, how? If worse, please also explain.
25. If the services are separate, how do people transfer from CAMHS to Working Age Adults and from Working Age Adults to Older Persons?
    (This is usually age cut-offs plus exceptions and complications. There seems to be huge variety in this and we would like to catalogue it.)

For peer review only - http://bmjopen.bmj.com/site/about/guidelines.xhtml

26. Does your service have a response time standard and is that time agreed with referrers and/or commissioners?

For peer review only

For peer review only - http://bmjopen.bmj.com/site/about/guidelines.xhtml

Appendix 2: Original and Modified criteria for describing Rapid Access Intervention and Discharge (RAID) services

Original RAID definition	• 24 hours, 7 days a week<br>• Age inclusive; no separate Older Age Adult or Working Age Adult teams<br>• Response targets of 1 hour to Emergency Department, 24 hours to wards<br>• Multidisciplinary team<br>• Comprehensive; see referrals for all clinical problems<br>• Brief follow-up clinics
Modified RAID definition	• 24 hours, 7 days a week<br>• Age inclusive; no dedicated Older Age Adult or Working Age Adult service<br>• Multidisciplinary team<br>• Response targets of 1 hour to Emergency Department , 24 hours to wards<br>• Either, not comprehensive (e.g. do not see substance misuse or self-harm referrals) or no follow-up clinics

For peer review only

Appendix 3: Core classifications according to Aitken et al, 2014. (13)

SubCore	Less Than Core
Core	2 consultants, 0.6 other medical,<br>2 band 7 nurses,<br>6 band 6 nurses,<br>0 other therapists<br>1 band 7 team manager,<br>0.2 band 8 clinical services manager<br>2.6 admins<br>9-5 hours<br>Sees everyone aged 16+
Core24	2 consultants, 2 other medical<br>6 band 7 nurses<br>7 band 6 nurses<br>4 other therapists<br>1 band 7 team manager<br>0.2-0.4 band 8 clinical services manager<br>2 admins<br>1 business support<br>24/7<br>Special older adults<br>Special Drugs and alcohol
Enhanced	4 consultants, 2 other medical<br>3 band 7 nurses<br>7 band 6 nurses<br>2 other therapists<br>1 band 7 team manager<br>0.2-0.4 band 8 clinical services manager<br>2 admins<br>1 business support<br>24/7<br>Special older adults<br>Special Drugs and alcohol<br>Outpatient services.
Comprehensive	5 consultants, 2 other medical<br>2 band 8b nurses<br>17 band 6 nurses<br>10 band 5 nurses<br>16 other therapists<br>3 band 7 team manager<br>1 band 8 clinical services manager<br>12 admins<br>1 business support<br>24/7<br>Special older adults<br>Special Drugs and alcohol<br>Outpatient services<br>Specialties

For peer review only - http://bmjopen.bmj.com/site/about/guidelines.xhtml

For peer review only

For peer review only - http://bmjopen.bmj.com/site/about/guidelines.xhtml

The Organisation and Delivery of Liaison Psychiatry Services in General Hospitals in England: results of a National Survey

Journal:	*BMJ Open*
Manuscript ID	bmjopen-2018-023091.R1
Article Type:	Research
Date Submitted by the Author:	03-Jul-2018
Complete List of Authors:	Walker, Andrew; National Institute of Health Research Clinical Research Network<br>Barrett, Jessica; Royal College of Psychiatrists, National Collaborating Centre for Mental Health<br>Lee, William; University of Plymouth, Peninsula Schools of Medicine & Dentistry<br>West, Robert; University of Leeds, Leeds Institute of Health Sciences<br>Guthrie, Elspeth; University of Leeds Leeds Institute of Health Sciences, Psychological and Social Medicine<br>Trigwell, Peter; Leeds and York Partnership NHS Foundation Trust, National Inpatient Centre for Psychological Medicine<br>Quirk, Alan; Royal College of Psychiatrists, CCQI<br>Crawford, Mike; Imperial College London, Psychological Medicine<br>House, Allan; University of Leeds Leeds Institute of Health Sciences
**Primary Subject Heading**:	Mental health
Secondary Subject Heading:	Health services research
Keywords:	MENTAL HEALTH, CONSULTATION-LIAISON, LIAISON PSYCHIATRY, HOSPITAL PSYCHIATRY, HEALTH SERVICES RESEARCH

SCHOLARONE™
Manuscripts

The Organisation and Delivery of Liaison Psychiatry Services in General Hospitals in England: results of a National Survey

Walker A[1]. A, Barrett J.R[2], Lee W[3], West R[4], Guthrie E[4], Trigwell P[5], Quirk A[6], Crawford M.J [6,7], House A[4]

1. Clinical Research Network National Coordinating Centre, National Institute of Health Research Clinical Research Network, Leeds
2. National Collaborating Centre for Mental Health, The Royal College of Psychiatrists
3. Institute of Translational and Stratified Medicine, Plymouth University Peninsula Schools of Medicine and Dentistry. Plymouth. UK.
4. Leeds Institute of Health Sciences, University of Leeds. Leeds. UK.
5. National Inpatient Centre for Psychological Medicine, Leeds and York Partnership NHS Foundation Trust. Leeds. UK.
6. College Centre for Quality Improvement. Royal College of Psychiatrists, London. UK.
7. Centre for Psychiatry. Department of Medicine. Imperial College London. London. UK.

Corresponding author: Professor Else Guthrie: e.a.guthrie@leeds.ac.uk

Key words: Consultation-liaison, hospital psychiatry, liaison psychiatry, health services research, mental health

Word Count :3943

Abstract: 298

For peer review only - http://bmjopen.bmj.com/site/about/guidelines.xhtml

ABSTRACT

Objectives: To describe current provision of hospital-based liaison psychiatry services in England, and to determine different models of liaison service that are currently operating in England.

Design: Cross-sectional observational study comprising an electronic survey followed by targeted telephone interviews.

Setting: All 179 acute hospitals with an Emergency Department in England.

Participants: 168 hospitals that had a liaison psychiatry service completed an electronic survey. Telephone interviews were conducted for 57 hospitals that reported specialist liaison services additional to provision for acute care.

Measures: Data included the location, service structures and staffing, working practices, relations with other mental health service providers, policies such as response times and funding. Model-2based clustering was used to characterise the services. Telephone interviews identified the range of additional liaison psychiatry services provided.

Results: Most hospitals (141, 79%) reported a 7-day service responding to acute referrals from the Emergency Department and wards. However, under half of hospitals had 24-hour access to the service (78, 44%). A third of hospitals (57, 32%) provided non-acute liaison work including outpatient clinics and links to specialist hospital services. 156 hospitals (87%) had a multidisciplinary service including a psychiatrist and mental health nurses. We derived a four-cluster model of liaison psychiatry using variables resulting from the electronic survey; the salient features of clusters were staffing numbers, especially nursing; provision of rapid response 24-hour 7-day acute services; offering outpatient and other non-acute work, and containing age-specific teams for older adults.

Conclusions: This is the most comprehensive study to date of liaison psychiatry in England, and demonstrates the wide availability of such services nationally. Although all services provide an acute assessment function, there is no uniformity about hours of coverage or expectation of response times. Most services were better characterised by the model we developed than by current classification systems for liaison psychiatry.

For peer review only - http://bmjopen.bmj.com/site/about/guidelines.xhtml

Article Summary

Strengths and limitations of this study

- A comprehensive national survey of liaison psychiatry services in acute hospitals, at a time of increased government investment, and debate about equity of access to mental and physical healthcare.
- The survey obtained 100% response rate for all hospitals in England with an Emergency Department.
- Classification of services was carried out using model-based clustering.
- A limitation was that service provision was reported by the services themselves rather than based on independent observation.
- Mental health services provided by clinicians outside of the liaison psychiatry service were not comprehensively reviewed.

For peer review only - http://bmjopen.bmj.com/site/about/guidelines.xhtml

For peer review only

INTRODUCTION

Liaison psychiatry is the sub-specialty of psychiatry concerned with clinical practice, teaching and research in non-psychiatric clinical settings: the 'liaison' referred to is therefore between psychiatry and other clinical disciplines. In the UK it has been largely based in acute ("general") hospitals. The origins of liaison psychiatry can be traced to the 1930s but substantial growth only occurred in the post-war decades (1-4): The UK's Royal College of Psychiatrists established its Faculty of Liaison Psychiatry in 1997 and first published a competency-based curriculum for postgraduate training in Liaison Psychiatry in 2009 (5).

The case for liaison psychiatry services rested initially upon observations that the prevalence of many psychiatric problems in acute hospitals is well above general population levels and that such co-morbidities can pose particular management challenges (6). People with problems such as psychosis, panic, delirium or self-harm may present to the Emergency Department, or their difficulties may become apparent on inpatient wards - perhaps requiring rapid assessment and intervention (7). Liaison psychiatry services also see people with more longstanding problems such as difficulty adjusting to severe physical illness, or complex physical health and mental health conditions – such work mostly being undertaken in outpatient clinics.

Recent interest in liaison psychiatry in the UK (8) has been focussed on two issues – cost savings that might result from the service, and the need to provide equitable access to emergency care for all patients regardless of whether their problems are primarily physical, psychiatric, or a combination (9).

The suggestion that financial savings from timely psychiatric intervention are sufficient to pay for the liaison psychiatry service undertaking that intervention, the so-called "cost-offset" effect, is not new (10). Most recently it attracted interest in the UK following the publication of a report from one English hospital which reported that their "Rapid Assessment Intervention and Discharge" (RAID) service achieved reductions in average inpatient lengths of stay in the target population of up to four days, even for patients not directly seen by the service (11) (12).

An important influence in current debate in the UK has been the classification of hospitals in terms of four service grades proposed by Aitken et al 2014 (13) (See Appendix for details). This

For peer review only - http://bmjopen.bmj.com/site/about/guidelines.xhtml

classification was based on services already in existence that were capable of delivering certain levels of coverage in the hospital. It has been used to inform commissioning of services, with the aim that all hospitals with Emergency Departments should have a liaison service meeting such standards by 2020 (14).

Here we present findings from the most detailed survey of liaison psychiatry services yet undertaken in England, describing their structures and staffing levels and their relation to other mental health services associated with the acute hospitals in which they are located. The aim was to describe current provision of hospital-based liaison psychiatry services in England, and to determine different models of liaison service that are currently operating.

This work arises from the first phase of a programme of research funded by the National Institute of Health Services (NIHR), to evaluate the cost-effectiveness and efficiency of different configurations of liaison psychiatry services in England (LP-MAESTRO) (http://www.nets.nihr.ac.uk/projects/hsdr/135808), and an annual mapping survey of liaison services funded by Health Education England, NHS England and the Royal College of Psychiatrists. A prior survey of liaison psychiatry services was carried out in 2013, and this paper describes the second study (15), which was carried out in conjunction with the LP-MAESTRO programme.

METHOD

Setting and sample

The sample consisted of all acute hospitals in England that had an Emergency Department at the time. Acute Trusts were identified from the NHS website (www.nhs.uk/servicedirectories/pages/nhstrustlisting.aspx) and individual hospitals were then identified from Trust websites.

Within each hospital liaison psychiatry service we identified *components* of service - typically defined by the part of the hospital covered by that component - for example: emergency department, ward referrals, links to specialist services, liaison psychiatry outpatient clinics.

Each component of the service might then have different *characteristics* such as staff mix, working hours, performance targets, patient-groups seen etc.

Design

Cross-sectional two-stage survey conducted by email and telephone interview.

Measures

The email survey ran between 14 May and 30 April 2015.The survey was brief and allowed flexible (free text) responses. Response was by email or telephone. Non-responding hospitals and missing response items were followed up by email and telephone. The questions asked in the email are given in Appendix 1.

We derived two variables describing RAID services. The first, "original RAID", is based upon the description provided in Tadros et al (12) of the service evaluated at Birmingham City Hospital; the second, "modified RAID", is based upon the profile of current services in Birmingham still known as

For peer review only - http://bmjopen.bmj.com/site/about/guidelines.xhtml

RAID. We characterised each service according to whether they met the criteria for either of these service types. We also used responses on staffing level or working practice to classify each service according to recent guidance from NHS England that was created to help commissioners in planning service delivery (13). The grades used in the guidance are as follows: Comprehensive (full liaison provision), Enhanced 24 (staffed according to the original RAID model), Core 24 (provides acute provision for a hospital with an Emergency Department, but no out-patient work) and Core (intended for less busy hospitals); services not meeting Core criteria were classified as Sub-Core (see Appendices 2 and 3 for details).

A telephone interview survey ran between the 16.7.15 and 30.9.15. It was undertaken to obtain further details about services that reported that they provided liaison services in addition to provision for acute care of patients in the ED or on the acute hospital wards (e.g. out-patient services or specialist renal input).

Data from the survey have been published by the Royal College of Psychiatrist (15). What we present in this paper is a re-classification of these data (carried out by the LP-MAESTRO team), a statistical analysis of the data using cluster analysis, and results of the telephone survey, none of which has been previously published.

Patient and participant involvement

Further work in the LP-MAESTRO programme will focus on patient experience. This will involve use of an on-line survey with service users (patients and carers) and non-psychiatric clinical staff who use liaison psychiatry services, with the aim of identifying additional outcomes and aspects of service that are not well characterised by quantitative work.

The results of the work presented in this paper will be disseminated to the liaison teams at each of the hospitals who took part in the study interviews.

ANALYSIS

The main analyses were undertaken with R statistical software version 3.2.2 (R core team 2016).

A latent class model (16) was fitted to perform clustering of responding hospitals. The number of clusters to be used was determined by minimising the Bayesian Information Criterion (BIC) because the BIC tends to favour less complexity. Models were fitted only if the number of observations (168) exceeded the number of parameters used in the model, thus ensuring a positive number of degrees of freedom. Other hospital properties were extracted from the survey and used as covariates in this model-based clustering approach.

Many of the variables used in clustering were categorical. Variables which might have been regarded as continuous were categorised so that all were handled in a similar way. For example, the number of hours of operation of the service was defined as three categories: 40–80 hours per week, 81–167 hours per week, and 168 (=7x24) hours per week. Since all variables to be clustered were categorical, the polytomous latent class analysis package poLCA version 1.4.1 (16) with R statistical software version 3.2.0 (R core team 2015) was used for all analyses. The latent class function made use of the Expectation–Maximisation algorithm and there was the possibility of convergence to a local maximum rather than a global maximum. To overcome this, multiple starts were used (17).

For peer review only - http://bmjopen.bmj.com/site/about/guidelines.xhtml

RESULTS

Staffing and working practices

Data were obtained on all 179 acute hospitals identified in England: 168 (94%) reported that they had a Liaison Psychiatry service; eleven had no service. All 168 hospitals with a liaison service completed the electronic survey and answered questions in follow up emails and telephone calls, ensuring that there were no missing data.

Twelve services were nurse-only services. All other services were multidisciplinary and all included at least a psychiatrist of some grade and a mental health nurse. One hundred and forty one hospitals (79%) reported at least one consultant psychiatrist as part of the team (total number =195), 95 hospitals (53%) reported other psychiatrists (non-consultant grade), 42 hospitals (23%) reported a psychologist or psychological therapist as part of the team, 26 hospitals (15%) reported allied health professionals and 52 hospitals (29%) reported other mental health staff. All 168 hospitals with a liaison service had nursing staff as part of the team and there were 1,384 whole time equivalents working in liaison services at the time of the survey.

141 hospitals (79%) provided a 7-day service and 15 hospitals (8%) provided a service Monday to Friday. Of the 141 hospitals providing 7-day services, 78 (55%) reported a 24-hour 7-day service.

Out of the 168 hospitals that had a liaison service, 75 hospitals (45%) had target response times of one hour or less for referrals from the Emergency Department and 73 hospitals (43%) reported target response times to referrals from the wards as less than one day. Sixty four hospitals (38%) had no target response time.

Nearly all of the liaison services (99%) saw patients who were referred following self-harm (167 hospitals) and many saw patients for assessment of alcohol and substance misuse (106 hospitals 63%). Only 37 services (22%) saw patients with learning disabilities. 44 services (26%) had separate older adult and working age adult teams.

57 hospitals (34%) reported a service or component of service that did more than serve the acute care pathway, and 4 of these hospitals operated virtually separate liaison services for acute and non-acute referrals.

Classification according to RAID and Core.

Of the 168 hospitals, only 8 met the original RAID criteria and 35 met criteria for modified RAID. Ten liaison services had the term RAID in their title, without meeting either of the RAID criteria.

Of the 168 hospitals, one was rated as Comprehensive, three were Enhanced24 (2%), 13 were Core24 (8%), 18 were Core (11%) and 133 were Sub-Core (79%). The Comprehensive rated service met modified RAID criteria, two of the Enhanced24 services met Original RAID criteria and the final Enhanced24 service did not meet either RAID criterion. Of those services that met either RAID criterion, 28/41 (68%) were rated as Core or Sub-Core.

Types of liaison psychiatry service: results from cluster analysis

We used data from the email survey to cluster the services using characteristics listed in Table 1.

For peer review only - http://bmjopen.bmj.com/site/about/guidelines.xhtml

The minimum value of Bayesian Information Criterion was with four clusters, but the value for three clusters was very near the minimum also. Hence a decision was required between three and four clusters and we decided the model with four clusters was more interpretable and useful. Hospitals were assigned to a cluster according to their modal probability: that is hospitals were labelled as a certain cluster when the model gave a probability of membership of that cluster to be larger than that of any other. Table 2 shows the modal cluster membership tabulated against hospital characteristics.

Model-based clustering identified four classes. These do not represent discrete categories but rather services that are relatively similar to each other in a diverse landscape.

- Cluster 1: Services tended to be based in smaller hospitals, had the smallest numbers of consultant staff and nurses. Only a minority offered 24-hour 7-day cover, few had predefined response times and none met either of the RAID criteria. Few offered outpatient clinics and none offered care outside the acute pathway.
- Cluster 2: Services were most likely to meet one of the RAID criteria, providing 24-hour 7-day cover, working to response-time targets for Emergency Department and ward referrals and concentrating exclusively on the acute care pathway with no follow-up outpatient clinics.
- Cluster 3: Services were more diverse with some offering 24-hour 7-day services, but the defining feature, was that they also offered outpatient clinics and covered care outside the acute care pathway; they had the highest number of consultants and nurses – number of nurses being an important determinant of the probability of membership in this cluster.
- Cluster 4: These were also diverse services, a third offering outpatient clinics and work outside the acute pathway; only a minority provided 24-hour 7-day cover or worked to response time targets and none met either of the RAID criteria. All these hospitals had separate teams for working-age adults and for older persons.

The nature of clinical services; telephone interviews

We undertook telephone interviews covering 57 hospitals and 61 separate liaison services; four hospitals had two distinctly different liaison teams. The telephone interviews reflected the clustering with most of those interviewed being in clusters 3 and 4: cluster 1: n=8, cluster 2: n=4, cluster 3: n=30 and cluster 4: n=19.

Emergency Department referrals

57 out of the 61 services (93%) saw acute referrals from the Emergency Department. Most (53, 87%) were available Monday to Sunday, and n=32 (52%) were available 24 hours a day. 49 (80%) of the services responded to referrals of adult patients of any age, but entry criteria could be quite specific – for example, one service saw all working age adults throughout the day and older age adults only for the first half of the night. Most Emergency Department liaison psychiatry teams (51, 84%) were multidisciplinary although five consisted of nursing staff only.

Referrals from the Emergency Department varied considerably in scope and numbers; out of the 46 reported referral rates the mean number of weekly referrals was 36 (min=1, max=100). In addition to assessment, most services that were interviewed (36, 59%) offered Emergency Department patients out-patient follow-up.

For peer review only - http://bmjopen.bmj.com/site/about/guidelines.xhtml

All of the 57 services which served the Emergency Department had key performance indicators, and almost three in four (n=43, 70%) measured patient outcome in some way.

Ward referrals

Fifty seven services accepted ward referrals. Most (n=46, 75%) were available to wards 7 days, and nearly one third (n=20, 33%) were available to wards 24 hours a day. Almost three out of four (n=44, 72%) responded to referrals from wards for adult patients of any age, five services responded to referrals from wards only for older adults, and nine responded to referrals from wards only for working age adults.

Again most ward teams (53, 87%) were multidisciplinary teams although four consisted of medical staff only, three consisted of nursing staff only, and one staffed the ward team with psychiatrists and psychologists. Based upon 47 reported referral rates the mean number of weekly referrals was 25. All responding services assessed patients and offered short term follow-up on medical wards, and over half (n=34, 56%) offered out-patient follow-up.

50 of those we interviewed had key performance indicators for wards, and 42 (69%) measured patient outcome in some way for ward referrals.

Self-harm referrals

All but two services accepted referrals for self-harm. 51 (84%) offered a 7 day service, 29 (48%) provided a service 24 hours a day, and most (n=46, 75%) offered a service to adults of all ages.

Most services we interviewed (54, 89%) said their self-harm teams were multidisciplinary. All services assessed patients on wards and approximately half (n=31, 51%) offered short-term follow-up on medical wards. Only eight services described a separate self-harm out-patient clinic, although several services described seeing small numbers of selected patients.

Liaison psychiatry with named specialist services

Twenty services (33%) provided specialist liaison services to at least one named specialist service or department in the hospital. A total of 31 different specialist services were reported; the most frequently reported were gastroenterology (n=5), hepatology (n=4) palliative care (n=4), maternity, neurology, trauma and transplant (n=3 each).

Outpatient clinics

33 services (54%) provided a general liaison psychiatry outpatient clinic and 28 services (46%) reported running an outpatient clinic for particular specialist groups; 20 services had both types of clinic. Twenty two of the general liaison psychiatry clinics saw patients of any adult age and 11 clinics saw working age adults only. Thirteen clinics were staffed with a multidisciplinary team, 15 were solely medical, two had nursing staff only and three clinics included a psychologist. Referrals to the general liaison psychiatry clinic came predominantly from the acute hospital in which they were based (n=26). Thirty one clinics offered short-term treatment and follow-up and 18 offered longer-term treatment and follow-up.

For peer review only - http://bmjopen.bmj.com/site/about/guidelines.xhtml

The most commonly reported specialist clinics were for medically unexplained symptoms (n=7), diabetes (n=4), bariatric surgery patients (n=4), respiratory disease (n=3) and perinatal psychiatry (n=3). Most clinics offered some form of psychological therapy – Problem Solving Therapy, (n=46, 75%) Motivational Interviewing, (n=38, 62%) Cognitive Behavioural Therapy (n=35, 57%) Behavioural Activation (n=31, 51%) or Interpersonal Therapy (n=26, 43%).

Other acute hospital mental health providers

In order of frequency, 47 liaison services (77%) co-existed in the acute hospital with separate drug or alcohol services, 44 (72%) with clinical psychology, and 22 (36%) co-existed with health psychology. We identified a wide range of other services – for particular patient groups or for overlapping patient groups by other agencies.

Referral to local service providers

The ease of referral to other mental health services for patients requiring follow up was also investigated. All services said they could routinely refer to a local community mental health team, 52 (85%) could refer to a crisis team routinely, 54 (89%) could refer routinely to drug and or alcohol and the same number to older adult psychiatry. Over half (n=36, 59%) of services could routinely refer to clinical psychology and 19 (31%) to health psychology.

Non-clinical activity

All services we interviewed provided some form of non-clinical work in the form of staff training or educational sessions, medico-legal assessments, advice to managers and others. The most common non-clinical services were: dementia training (n=17); research and service evaluation (n=16); organizational support and advice to acute hospital staff (n=15); delirium training (n=11); Mental Capacity Act training (n=10).

Discussion

We identified widespread availability of liaison psychiatry services in acute hospitals in England. Liaison psychiatry teams were customarily multidisciplinary and most services saw all acute mental health problems in the hospital and adults of all ages. Our findings suggest that there has been a gradual but continued expansion in liaison psychiatry services over the last 20 years, as evidenced by several previous surveys including those focusing on consultant posts in the British Isles (18,19), services in a particular area of England (20) and the one previous unpublished national survey undertaken at the request of NHS-England (LPSE-1). As an example of the expansion, the number of consultant posts in liaison psychiatry in the British Isles more than doubled from 43 in 1998 (18) to 93 in 2003 (19). The findings of the current survey suggest a further increase with 195 consultant posts in liaison psychiatry in England alone.

For peer review only - http://bmjopen.bmj.com/site/about/guidelines.xhtml

We found 11 hospitals that reported having no liaison service at all, which is concerning given that one of the targets set by NHS England in the Five Year Forward View for Mental Health is that by 2020 no acute hospitals should be without all-age mental health liaison services in emergency departments and inpatient wards (21).

Only a third of services offered outpatient clinics and non-acute care. The range of such activities was wide, with more than 30 different specialist services. There was however very little in common between services about which specialist activities were supported. Surprisingly few services (just over 10% of our total sample) reported running clinics that supported longer-term follow up and treatment opportunities, a *sine qua non* for the management of problems with living with long-term illness or of severe and chronic medically unexplained symptoms (22). This gap in service provision was most striking in self-harm services; we found that the majority of services offered acute assessment but no service offered routine therapeutic treatment for service users.

Very few of the services we surveyed readily fitted into the current commissioning framework (13), or the RAID framework, so the classification into Core, Core24, Enhanced24, or Comprehensive had limited value in discriminating between hospitals, and neither descriptive framework proved useful in identifying those services that reported 24-hour 7-day acute services.

For these reasons we sought a more practical, data-driven approach to describing service types. We chose model-based clustering to do so. Alternative approaches would have been to use one of many heuristic algorithms such as hierarchical clustering, k-means, self-organising maps, graph-theoretic approaches, or support vector machines. A generative mixture model has the advantage that it can be more general and provides a statistical framework within which to decide upon the number of clusters present. Model-based clustering has also been found to perform better than other approaches in identifying clusters (16). The models were simply parameterised since there were only 168 observations. Within a diverse picture of provision, our cluster analysis did reveal some patterns of service – the three most obvious features that distinguished between services were the hours of cover and response time standards, the likelihood of providing non-acute care in outpatients, and the decision to have separate teams for older and working age adults. Size of hospital and staffing levels (especially nursing) were important associations with the type of service offered. This suggests that when services scale up from the basic provision represented by Cluster 1 (and found in smaller hospitals) they do so in one of these three directions – increasing intensity of acute work, developing outpatient and non-acute work, or developing specialist old age teams.

Our findings have implications for those commissioning and those providing services.

First, we found widespread availability of liaison psychiatry services in English acute hospitals, but most teams were poorly resourced compared to published recommendations. Second, whatever local decisions are made about liaison psychiatry, our survey suggests national co-ordination of services is lacking. Third, we were struck by the unexpectedly low levels of longer-term outpatient treatment provision. Problems of adjustment to long-term illness, persistently poor adherence to challenging treatment regimes, medically unexplained symptoms and severe somatoform disorders all form part of the *raison d'etre* for liaison psychiatry and their management requires sustained

For peer review only - http://bmjopen.bmj.com/site/about/guidelines.xhtml

professional input, in the hospital as well as in community settings. Our results confirm previous findings about the low national level of provision for people who harm themselves.

There are several limitations to our study. Our approach to surveying provided a rather general high-level account of services that doesn't do full justice to the richness and diversity of provision in multi-component services. Reliance on a single (or occasionally a second) informant at each stage may have led to missing or inaccurate information. The service descriptors we used were based on self-report, and we have not verified them with direct independent observation. Our sampling strategy meant we did not collect information on specialist hospitals without Emergency Departments, so we did not collect data on rare but important facilities in specialist hospitals. Our survey was entirely hospital focused and while we are aware of (and involved in) initiatives to develop and evaluate primary care-based liaison psychiatry services, they were not studied here.

There is increasing interest in the idea that well-run liaison psychiatry services can be both important in improving quality of care in acute hospitals and cost-effective (23-25). UK liaison services are changing rapidly, with a round of investment especially in provision for emergency assessment and response (26). A further national survey of all English acute hospitals has been completed and the results will be published in the near future. It is hoped this latest survey will provide further coverage of a rapidly changing landscape.

Liaison psychiatry services in the UK are being encouraged by their specialty representative group in the Royal College of Psychiatrists to use a standardised package of outcome measures, the Framework for Routine Outcome Measurement in Liaison Psychiatry (FROM-LP) (27), to enable benchmarking against national norms. On the basis of these and other evaluation exercises we expect to achieve an increasingly detailed and nuanced account of the nature and impact of liaison psychiatry – a subspecialty that has a valuable role in providing genuinely co-ordinated and inclusive healthcare.

For peer review only

For peer review only - http://bmjopen.bmj.com/site/about/guidelines.xhtml

Acknowledgments: This work was supported by the efforts of S Keane, M Heneghan, S Chee, J Lewis, S Jayakumar and H Lucas at the Royal College of Psychiatrists and the NIHR Clinical Research Network (CRN) Yorkshire and Humber. J Hewison, C Czoski Murry, C Smith, C Hulme, S Tubeuf, A Martin, S Relton from the Leeds Institute of Health Sciences and Matthew Fossey from the Centre for Mental Health Research are fellow collaborators on the programme.

Funding: The present study arises from two funding initiatives: A national survey of staffing and structure in liaison psychiatry services in acute hospitals completed on behalf of the Royal College of Psychiatrists and the National Collaborating Centre for Mental Health commissioned by NHS England (Liaison Psychiatry Survey of England 2015, LPSE 2015) and a research study funded by the UK's National Institute for Health Research HS&DR programme to evaluate the effectiveness and cost-effectiveness of liaison psychiatry services (LP-MAESTRO project number 13/58/08).

Department of Health Disclaimer: The views and opinions expressed therein are those of the authors and do not necessarily reflect those of the HS&DR, NIHR, NHS or the Department of Health.

Author Contributions: AH, MJC, EG, and PT conceived of the research. JRB and WL carried out the survey. AW co-ordinated the telephone interviews. RW carried out the statistical analysis. AQ and all other authors contributed to the manuscript and approved the final version.

Provenance and peer review: Not commissioned; externally peer reviewed.

Competing Interests: All authors have completed the ICMJE uniform disclosure form at http://www.icmje.org/coi_disclosure.pdf and declare: no support from any organization for the submitted work; no financial relationships with any organizations that might have an interest in the submitted work in the previous three years, no other relationships or activities that could appear to have influenced the submitted work.

Ethical approval and consent

Return of the email survey was taken as implicit consent. NHS Ethical permission (REC reference: 15/NS/0025) and Trust level approval was obtained for the telephone interviews. Telephone survey interviewees provided verbal consent at the start of the interview.

Data Sharing

Survey data are available from the author WL.

For peer review only - http://bmjopen.bmj.com/site/about/guidelines.xhtml

References

1. Lipowski Z. Consultation-liaison psychiatry: the first half century. General hospital psychiatry. 1986;8(5):305-15.
2. Mayou R. The history of general hospital psychiatry. The British Journal of Psychiatry. 1989;155(6):764-76.
3. Schwab JJ. Consultation-liaison psychiatry: a historical overview. Psychosomatics. 1989;30(3):245-54.
4. Aitken P, Lloyd G, Mayou R, Bass C, Sharpe M. A history of liaison psychiatry in the UK. BJPsych Bull 2016; 40: 199-203.
5. The Royal College of Psychiatrists. A competency-based curriculum for specialist training in psychiatry: sub-specialist module in liaison psychiatry. RCPsych London. 2006.
6. Bourgeois J HD, Servis M, Hales R. Consultation-liaison psychiatry: advantages for healthcare systems. Disease Management and Health Outcomes. 2005;13(2):93-106.
7. Academy of Medical Royal Colleges. Managing Urgent Mental Health needs in the Acute Hospital: a guide for practitioners, managers and commissioners. London 2009.
8. Joint Commissioning Panel for Mental Health.. Guidance for commissioners of liaison mental health services to acute hospitals. London. 2012.
9. Parsonage M, Fossey M, Tutty C. Liaison psychiatry in the modern NHS. London: Centre for Mental Health. 2012.
10. Strain JJ, Hammer JS, Fulop G. APM task force on psychosocial interventions in the general hospital inpatient setting: a review of cost-offset studies. Psychosomatics. 1994;35(3):253-62.
11. Parsonage M, Fossey M. Economic evaluation of a liaison psychiatry service: Centre for Mental Health London; 2011.
12. Tadros G, Salama RA, Kingston P, Mustafa N, Johnson E, Pannell R, et al. Impact of an integrated rapid response psychiatric liaison team on quality improvement and cost savings: the Birmingham RAID model. The Psychiatrist Online. 2013;37(1):4-10.
13. Aitken P, Robens S, Emmens T. Developing Models for Liaison Psychiatry Services-Guidance. Strategic Clinical Network for Mental Health, Dementia and Neurological Conditions South West. England 2014.
14. Department of Health, England. Achieving Better Access to Mental Health Services by 2020. London. 2014.
15. Barrett J, Aitken P, Lee W. Report of the 2nd Annual Survey of Liaison Psychiatry in England. Royal College of Psychiatrists https://www.rcpsych.ac.uk/pdf/CALC_LIA2015WilliamLee1.pdf Retrieved Dec 12 2017

16. Linzer DA, Lewis JB. poLCA: An R package for polytomous variable latent class analysis. Journal of Statistical Software. 2011;42(10):1-29.

17. Yeung KY, Fraley C, Murua A, Raftery AE, Ruzzo WL. Model-based clustering and data transformations for gene expression data. Bioinformatics. 2001;17(10):977-87.

18. Guthrie E Development of liaison psychiatry: Real expansion or a bubble that is about to burst. BJPsych Bulletin 1998; 22:291-293.

19. Swift G, Guthrie E. Liaison psychiatry continues to expand: developing services in the British Isles. BJPsych Bulletin 2003; 27: 339-341.

For peer review only - http://bmjopen.bmj.com/site/about/guidelines.xhtml

20. Ruddy R, House A. A standard liaison psychiatry service structure? The Psychiatrist. 2003;27(12):457-60.

21. Mental Health Taskforce Strategy. The Five Year Forward View for Mental Health. A report from the independent Mental Health Taskforce to the NHS in England. Feb 2016 www. nhs.uk/mentalhealth/taskforce

22. Creed FH, van der Feltz-Cornelius C, Guthrie E, Henningsen P, Rief W, Schroder A, White P. Identification, assessment and treatment of individual patients. Chapter in Medically Unexplained Symptoms, Somatisation and Bodily Distress: Developing better clinical services. Eds F Creed P Henningsen & P Fink. Cambridge University Press. 2011 p175-216.

23. Wood R, Wand AP. The effectiveness of consultation-liaison psychiatry in the general hospital setting: a systematic review. Journal of psychosomatic research. 2014;76(3):175-92.

24. de Araujo Andreoli PB, de Albuquerque Citero V, de Jesus Mari J. A systematic review of studies of the cost-effectiveness of mental health consultation-liaison interventions in general hospitals. Psychosomatics. 2003;44(6):499-507.

25. Hosaka T, Aoki T, Watanabe T, Okuyama T, Kurosawa H. General hospital psychiatry from the perspective of medical economics. Psychiatry and clinical neurosciences. 1999;53(4):449-53.

26. NHS England . Guidance to support the introduction of access and waiting time standards for mental health services in 2015/16. https://www.england.nhs.uk/wp-content/uploads/2015/02/mh-access-wait-time-guid.pdf Retreived 10 Dec 2017.

27. Trigwell P, Kustow J, Santhouse A, et al. *Framework for Routine Outcome Measurement in Liaison Psychiatry (FROM-LP).* Faculty Report FR/LP/02. Royal College of Psychiatrists, 2015.

For peer review only

For peer review only - http://bmjopen.bmj.com/site/about/guidelines.xhtml

Table 1: The characteristics derived from survey responses used to distinguish Liaison Psychiatry services in the model based clustering

**Labelling**	1. Does the name of the service include 'RAID'?<br>2. Is the service classified as Sub-core, Core, or does it meet one of the definitions Core 24, Enhanced, or Comprehensive?<br>3. Does the service operate 7 days per week, or for less than 7 days?<br>4. How many hours per week is the service provided?
**Coverage**	5. Does the service claim to cover all mental health?<br>6. Is there a dedicated working-age adults (18–65) team?<br>7. Is there a dedicated older adults (65+) team?
**Work done**	8. Does the service undertake work from the Emergency Department?<br>9. Does the service undertake in-reach work?<br>10. Does the service operate an out-patient clinic?<br>11. Does the service have pathways other than acute pathways?<br>12. What is the response time for the Emergency Department?<br>13. What is the response time for the wards?
Other aspects of hospitals (Note that for some variables, the value assigned may have been inferred from other survey responses rather than taken from the direct response given.)	14. Number of services within a hospital (1,2, or 3)<br>15. Number of providers of services (1 or 2)<br>16. Number of hospital beds<br>17. Number of nurses employed by the liaison psychiatry service<br>18. Number of consultants<br>19. Number of services<br>20. Number of service providers
Additional variables	21. Does the service meet the original RAID criteria?<br>22. Does the service meet the modified RAID criteria?

RAID = Rapid Assessment Intervention and Discharge

For peer review only - http://bmjopen.bmj.com/site/about/guidelines.xhtml

Table 2: Hospital characteristics according to cluster membership

Hospital characteristic	Cluster 1 N=46	Cluster 2 N=35	Cluster 3 N=43	Cluster 4 N=44
RAID				
Name has RAID in title	1 (2%)	7 (20%)	10 (23%)	1 (2%)
Not codable for RAID	3 (6%)	1 (3%)	5 (12%)	1 (2%)
Not RAID	43 (94%)	11 (31%)	20 (46%)	43 (98%)
Original RAID	0 -	0 -	6 (14%)	0 -
Modified RAID	0 -	23 (66%)	12 (28%)	0 -
Core classification				
Sub-Core	45 (98%)	23 (66%)	29 (67%)	36 (82%)
Core	1 (2%)	5 (14%)	7 (16%	5 (11%)
Core 24	0 -	7 (20%)	3 (7%)	3 (7%)
Enhanced24	0 -	0 -	3 (7%)	0 -
Comprehensive	0 -	0 -	1 (2%)	0 -
Service operates 7 days	33 (72%)	34 (97%)	34 (79%)	40 (91%)
Hours of Operation				
40–80 hours	15 (33%)	1 (3%)	8 (19%)	15 (34%)
81–167 hours	16 (34%)	7 (20%)	13 (30%)	15 (34%)
7x24 = 168 hours	15 (33%)	27 (77%)	22 (51%)	14 (31%)
Serves all MH	32 (70%)	21 (60%)	26 (60%)	19 (43%)
Dedicated WAA	0 -	0 -	0 -	44 (100%)
Dedicated OAA	0 -	0 -	0 -	44 (100%)
OP clinic	2 -	0 -	43 (100%)	14 (32%)
Non-acute pathway	0 -	0 -	43 (100%)	14 (32%)
Response time to the ED				
<1h	3 (6%)	35 (100%)	25 (58%)	12 (27%)
1.5–4h	17 (37%)	0 -	4 (9%)	11 (25%)
Not stated or >4h	26 (57%)	0 -	14 (33%)	21 (48%)
Response time to wards				
<24h	8 (17%)	29 (83%)	23 (53%)	13 (30%)
36h–5d	8 (17%)	6 (17%)	0 -	8 (18%)
Not stated	30 (65%)	0 -	20 (46%)	23 (52%)
Hospital beds				
50–447	19 (41%)	14 (40%)	12 (28%)	8 (18%)
447–621	20 (43%)	8 (24%)	16 (37%)	16 (36%)
622–1943	7 (15%)	13 (54%)	17 (40%)	20 (45%)
Number of FTE nurses				
0.5–6.0	25 (54%)	8 (23%)	11 (26%)	16 (36%)
6.1–9.4	15 (33%)	11 (31%)	11 (26%)	15 (34%)
9.5–24.0	6 (13%)	16	21 (49%)	13 (30%)
Number of FTE consultants				
0.0–0.5	27 (57%)	7 (20%)	9 (21%)	15 (34%)
0.6–1.4	16 (35%)	13 (37%)	12 (28%)	13 (30%)
1.5–9.2	3 (6%)	15 (43%)	22 (51%)	16 (36%)
Single service provider	45 (98%)	33 (94%)	41 (95%)	40 (91%)
Two service providers	1 (2%)	2 (6%)	2 (5%)	4 (9%)
Number of services within same hospital				
One	44 (96%)	35 (100%)	39 (91%)	27 (61%)
Two	1 (2%)	0 -	4 (9%)	16 (36%)
Three	1 (2%)	0 -	0 -	1 (2%)

MH Mental Health WAA= Working Age Adults OAA =Older Adults Service FTE= Full time equivalent

For peer review only - http://bmjopen.bmj.com/site/about/guidelines.xhtml

On-line appendices for paper entitled "The Organisation and Delivery of Liaison Psychiatry Services in General Hospitals in England: results of a National Survey".

Appendix 1: Questions used in the Second National Liaison Psychiatry Survey (LPSE-2) in 2015.

Questions 1-10: Location

1. What is the name of your Liaison Psychiatry service (if it has one)?
2. What is the name of the Acute Hospital(s) you are based in?
3. What is the name of the Acute Trust(s) you are based in?
4. Does the Acute Trust(s) have more than one site with inpatient beds? If so, please name them.
5. Does the Acute Trust(s) have more than one A&E? If so, please name them.
6. Does your Liaison Psych service provide services to all the sites?
7. If not, can you give us a contact details of the other liaison psychiatry service(s) please?
8. What is the provider of your service? (Usually this is the mental health trust)
9. Is psych liaison in your Acute Trust provided by one or many providers? If many, which?

If the above questions do not capture details of your service, please explain here:

Questions 11-12: Target population

10. What services do you provide, and to whom? (Some only see self-harms, some see anyone in the whole hospital, others are in-between. Some look after alcohol problems, some not, some do LD, some not, etc.) What are the age-criteria for your service(s)?
11. Do you support anything other than the acute care pathway? Are there any clinics, etc. If so, can you outline the nature of the work?

Questions 13-18: Staffing

12. Number of FTE nurses and their bands (if working age adults and older adults are separate services, please collate these separately)
13. Number of FTE doctors and their grades (if working age adults and older adults are separate services, please collate these separately).
14. Number of FTE admins and their grades (if working age adults and older adults are separate services, please collate these separately).
15. Number of other clinicians and their grades if known (if working age adults and older adults are separate services, please collate these separately).
16. Number of other non-clinicians and their grades if known (if working age adults and older adults are separate services, please collate these separately).
17. Of the above, who is substantive and who is a locum, part of winter pressures. fixed term appointments, etc?

Questions 19-20: funding

18. What is your service's budget, if known? (Leave out the medics (or just junior medics) if necessary).
19. How much of that that budget is permanent and how much is temporary (if known)?

Questions 21-23: Mental health service context

20. What are your service's hours of operation? (Out Of Hours SHO cover does not mean your service is 24/7).
21. Does your service do all the work contained in all the referrals? (eg is some passed on to other services? Please explain)

    (This question is about things like requests for psych opinions from wards, which are sometimes passed straight on to the duty SHO)
22. Are there other mental health workers in your acute trust who are not part of your service? (eg counsellors, psychologists)

Questions 24-28: Commissioning context

23. Have you undertaken any research (published or not) to support the development of your service? If so, can you describe it please?
24. Is your service better resourced than it was a year ago? If so, how? If worse, please also explain.

For peer review only

25. If the services are separate, how do people transfer from CAMHS to Working Age Adults and from Working Age Adults to Older Persons?
(This is usually age cut-offs plus exceptions and complications. There seems to be huge variety in this and we would like to catalogue it.)

26. Does your service have a response time standard and is that time agreed with referrers and/or commissioners?

For peer review only

For peer review only - http://bmjopen.bmj.com/site/about/guidelines.xhtml

Appendix 2: Original and Modified criteria for describing Rapid Access Intervention and Discharge (RAID) services

Original RAID definition	• 24 hours, 7 days a week<br>• Age inclusive; no separate Older Age Adult or Working Age Adult teams<br>• Response targets of 1 hour to Emergency Department, 24 hours to wards<br>• Multidisciplinary team<br>• Comprehensive; see referrals for all clinical problems<br>• Brief follow-up clinics
Modified RAID definition	• 24 hours, 7 days a week<br>• Age inclusive; no dedicated Older Age Adult or Working Age Adult service<br>• Multidisciplinary team<br>• Response targets of 1 hour to Emergency Department , 24 hours to wards<br>• Either, not comprehensive (e.g. do not see substance misuse or self-harm referrals) or no follow-up clinics

For peer review only

Appendix 3: Core classifications according to Aitken et al, 2014. (13)

SubCore	Less Than Core
Core	2 consultants, 0.6 other medical,<br>2 band 7 nurses,<br>6 band 6 nurses,<br>0 other therapists<br>1 band 7 team manager,<br>0.2 band 8 clinical services manager<br>2.6 admins<br>9-5 hours<br>Sees everyone aged 16+
Core24	2 consultants, 2 other medical<br>6 band 7 nurses<br>7 band 6 nurses<br>4 other therapists<br>1 band 7 team manager<br>0.2-0.4 band 8 clinical services manager<br>2 admins<br>1 business support<br>24/7<br>Special older adults<br>Special Drugs and alcohol
Enhanced	4 consultants, 2 other medical<br>3 band 7 nurses<br>7 band 6 nurses<br>2 other therapists<br>1 band 7 team manager<br>0.2-0.4 band 8 clinical services manager<br>2 admins<br>1 business support<br>24/7<br>Special older adults<br>Special Drugs and alcohol<br>Outpatient services.
Comprehensive	5 consultants, 2 other medical<br>2 band 8b nurses<br>17 band 6 nurses<br>10 band 5 nurses<br>16 other therapists<br>3 band 7 team manager

For peer review only - http://bmjopen.bmj.com/site/about/guidelines.xhtml

	1 band 8 clinical services manager<br>12 admins<br>1 business support<br>24/7<br>Special older adults<br>Special Drugs and alcohol<br>Outpatient services<br>Specialties

For peer review only

For peer review only - http://bmjopen.bmj.com/site/about/guidelines.xhtml

Title and abstract	1	a) Title describes the design of the study-i.e. survey- The Organisation and Delivery of Liaison Psychiatry Services in General Hospitals in England: results of a National Survey<br>b) Abstract provides a balanced summary of the study and its findings
Introduction		
Background and rationale	2	Scientific background explained-introduction -first 5 paragraphs
Objectives	3	Objectives stated, no sub-analyses. Introduction Paragraph 6- The aim was to describe current provision of hospital-based liaison psychiatry services in England, and to determine different models of liaison service that are currently operating in England.
Methods		
Study design	4	Design- Cross-sectional two-stage survey conducted by email and telephone interview.
Setting	5	Setting is described in detail. All acute hospitals in England with an emergency department. No follow-up.
Participants	6	Two stage design: first stage survey of liaison psychiatry services in acute hospitals in England. Second stage:telephone survey involving liaison psychiatry staff at services which provide more than ‘acute cover’. Intention to obtain more detailed understanding of what additional services were available at these Trusts.
Variables	7	No formal measures were used. Details of liaison services that were included in the survey are listed in the appendix.
Data sources/measurement	8	See above. Data sources were the responses to the questionnaire survey (stage one) and to the telephone interviews (staged two)
Bias	9	Our approach to surveying provided a rather general high-level account of services that doesn’t do full justice to the richness and diversity of provision in multi-component services.<br>Reliance on a single (or occasionally a second) informant at each stage may have led to missing or inaccurate information.<br>The service descriptors we used were based on self-report, and we have not verified them with

For peer review only

		direct independent observation. Our sampling strategy meant we did not collect information on specialist hospitals without Emergency Departments, so we did not collect data on rare but important facilities in specialist hospitals.<br>The survey was entirely hospital focused and while we are aware of (and involved in) initiatives to develop and evaluate primary care-based liaison psychiatry services, they were not studied here.
Study size	10	All hospitals in England with an ED that had a liaison psychiatry service n=168
Quantitative variables		Many of the variables used in clustering were categorical. Variables which might have been regarded as continuous were categorised so that all were handled in a similar way. For example, the number of hours of operation of the service was defined as three categories: 40–80 hours per week, 81–167 hours per week, and 168 (=7x24) hours per week.
Statistical methods		a) A latent class model (15) was fitted to perform clustering of responding hospitals. The number of clusters to be used was determined by minimising the Bayesian Information Criterion (BIC) because the BIC tends to favour less complexity. Models were fitted only if the number of observations (168) exceeded the number of parameters used in the model, thus ensuring a positive number of degrees of freedom. Other hospital properties were extracted from the survey and used as covariates in this model-based clustering approach.<br>Since all variables to be clustered were categorical, the polytomous latent class analysis package poLCA version 1.4.1 (Linzer and Lewis 2011) with R statistical software version 3.2.0 (R core team 2015) was used for all analyses. The latent class function made use of the Expectation–Maximisation algorithm and there was the possibility of convergence to a local maximum rather than a global maximum. To overcome this multiple starts were used.<br>There were no missing data.<br>There were no sensitivity analyses
Results		
Participants	13	Responses from all eligible hospitals n=168
Descriptive data	14	Data are provided in results section under 'staffing and working practices'.

For peer review only

		No missing data
Outcome data	15	Main outcome data are shown in Table 2 accompanying paper. Data are presented as number and percentages
Main results	16	Survey data are represented as number and percentages.
Other analyses	17	No sensitivity analyses. Cluster analysis is described in detail in statistics section.
Discussion		
Key results	18	Key results are presented with reference to study objectives.
Limitations	19	Discussed full in results section-paragraph 6
Interpretation	20	Results are interpreted cautiously
Generalisability	21	Limited to hospitals in England with emergency departments.
Other information		
Funding	22	Source of funding is stated 'The present study arises from two funding initiatives: A national survey of staffing and structure in liaison psychiatry services in acute hospitals completed on behalf of the Royal College of Psychiatrists and the National Collaborating Centre for Mental Health commissioned by NHS England (Liaison Psychiatry Survey of England 2015, LPSE 2015) and a research study funded by the UK's National Institute for Health Research HS&DR programme to evaluate the effectiveness and cost-effectiveness of liaison psychiatry services (LP-MAESTRO .project number 13/58/08).'

For peer review only
